# Children’s participation in assent to paediatric oncology research: experiences of children, siblings and parents

**DOI:** 10.1186/s13063-026-10011-2

**Published:** 2026-09-21

**Authors:** Kajsa Norberg Wieslander, Sara Frygner-Holm, Stefan Nilsson, Malin Lövgren, Anna Höglund, Tove Godskesen

**Affiliations:** 1 Centre for Research Ethics and Bioethics, Department of Public Health and Caring Sciences, Uppsala University, Uppsala, Sweden; 2 Department of Women’s and Children’s Health, Uppsala University, Uppsala, Sweden; 3 Institute of Health and Care Sciences, University of Gothenburg, Gothenburg, Sweden; 4 Centre for Person-Centred Care (GPCC), Sahlgrenska Academy, Institute of Health and Care Sciences, University of Gothenburg, Gothenburg, Sweden; 5 Sahlgrenska University Hospital, Queen Silvia Children’s Hospital, Region Västra Götaland, Gothenburg, Sweden; 6 Department of Health Care Sciences, Marie Cederschiöld University, Stockholm, Sweden; 7 Faculty of Nursing and Health Sciences, Nord University, Bodø, Norway

**Keywords:** Ethics, Research, Informed consent, Assent, Children, Paediatric oncology

## Abstract

**Background:**

Research is essential for improving outcomes in paediatric oncology, yet decisions about research participation often occur during emotionally vulnerable periods for families. Although children’s assent is ethically required, it is not always meaningfully implemented in practice. Understanding how children, siblings and parents experience the child’s involvement in the assent process, including their attitudes towards research, is crucial for promoting ethically sound research and supporting genuine child participation. The aim of this study was to describe how children with cancer, siblings and parents experience the child’s assent process during research recruitment in paediatric oncology, with particular attention to its motivational and relational dimensions.

**Methods:**

A descriptive qualitative study design. Interviews were undertaken with children with cancer (*n* = 11), siblings (*n* = 17) and parents (*n* = 9) regarding their experiences of recruitment to various types of research in Sweden. Data was analysed using reflexive thematic analysis.

**Results:**

Two main themes were identified: *a moral and emotional commitment to research*, and *relational assent—trust, loyalty and parental mediation.* Children, siblings and parents described research participation as meaningful and morally significant in a vulnerable situation. Children’s involvement in assent ranged from active participation to limited influence, with some decisions made primarily by parents. Decisions about research participation were shaped by relational dynamics, in which trust, loyalty, and emotional vulnerability and parental mediation played central roles.

**Conclusions:**

Families are strongly motivated to contribute to research in paediatric oncology. However, sustaining trust and safeguarding research integrity require consistent and meaningful implementation of children’s assent. Child-centred, flexible, and context-sensitive assent processes are key, alongside ethical awareness of how relational dynamics shape children’s autonomy. Recognising parents as key partners is essential for upholding both ethical standards and children’s rights in paediatric research.

**Supplementary Information:**

The online version contains supplementary material available at https://doi.org/10.1186/s13063-026-10011-2.

## Background

Research is essential to improve survival, treatment strategies, and quality of life for children with cancer. Owing largely to advances achieved through clinical research, survival rates now exceed 80% in many European countries [1]. As a result, many families are asked to consider participation in research and to provide consent [2]. However, recruitment in paediatric oncology raises significant ethical challenges [3]. Psychological distress at diagnosis, time pressure and limited understanding of complex study information may complicate the informed consent process [4]. A child’s cancer diagnosis affects the entire family, including parents and siblings, who are increasingly involved not only in care but also as research participants [5, 6]. Given the vulnerable and emotional strain experienced by these families, careful attention to the ethical dimensions of research recruitment is essential [7].

Ethical guidelines for research involving children who cannot legally consent, emphasise the importance of children’s own assent—a process in which children actively and voluntarily agree to participate in research based on adapted information and mutual dialogue [8, 9]. Assent is generally recommended from around seven years although not considered an age threshold, as all capable children should be engaged in assent [10]. In Sweden, clinical research involving children is governed by the Ethical Review Act and additional regulations, summarised in Table 1. Together, these frameworks establish that children should receive age-appropriate information, be given the opportunity to express their views, and that their objections must be respected.

**Table 1:** Relevant regulations for children’s participation and assent to research in Sweden

Swedish Ethical Review Act (2003:460). Parents must provide informed consent for children under 15 years of age. Children should also receive age-appropriate information and give their assent, if capable. Even with parental consent, research cannot proceed if a child under 15 understands the implications and objects. Children must have the chance to express objections, particularly when they are in dependent relationships with researchers or may face challenges protecting their rights [11]. Additional legislation applies to clinical drug trials, medical device studies, and biobank research in accordance with EU regulations. Ethical review across these frameworks is based on common principles, including respect for human dignity, proportional risk–benefit assessment, and the protection of participants’ rights and well-being
United Nations Convention on the Rights of the Child (UNCRC). Legally binding in Sweden, the UNCRC emphasises a child’s right to actively participate in decisions affecting them. For example, Article 12 states that children who are capable of forming their own views have the right to freely express them, and that their views should be given appropriate weight based on their age and maturity [12]
Swedish Patient Act (2014:821). This Act requires healthcare professionals to consider children’s healthcare-related preferences and give them due weight, and obligates the consideration of a child’s best interests in decisions concerning the child’s health [13]
Parental legal responsibilities. According to Swedish parental code and the UNCRC, parents are obligated to provide a good upbringing that supports their children’s development and respects them as individuals. Parental decisions must prioritise the child’s best interests [14]

These legal and ethical requirements place demands on child-centred and flexible research practices [8, 9]. Today, there is an increased emphasis on ensuring that assent is grounded in *meaningful* participation. According to the Council of Europe [15], this involves participation that is respectful, ethical, and constructive; based on age-appropriate information, safe and inclusive environments where children feel able to express their views, as well as the responsibility of parents and professionals to protect and safeguard the child’s best interests. A child-centred approach recognises children as independent rights-holders and active participants within the relational context of their family [16, 17]. As children’s decision-making capacity is emerging and variable, a balance is required between participation and protecting the child from burdens, risks, including coercion and undue influence in research recruitment, through safeguarding mechanisms such as the requirement for parental permission [9, 18].

Importantly, children—including school-aged children—will typically have views about what matters to them [19], and when adequately supported, can engage meaningfully in decisions about research participation [20]. Nevertheless, evidence suggests that children are not consistently involved in assent processes. A recent Nordic study found that one-third of school-aged children with cancer aged 8 years or older, were not involved in assent processes [21], consistent with previously reported trends [22, 23]. Participation and being informed have been associated with positive aspects for the child, including a sense of control, preparedness, and trust in adults [24]. In research, assent represents a morally significant practice grounded in respect for the child’s emerging autonomy, integrity and personhood [25].

Previous empirical research on this topic has primarily focused on the perspectives of healthcare professionals and parents [7, 21, 23, 26]. Studies exploring children’s own recruitment experiences have often centred on adolescents or broad age ranges, making it difficult to discern the specific experiences of assent-aged children (< 15 years) [27–29].

Moreover, research involving siblings has addressed experiences of participation rather than the assent process itself [30, 31]. Thus, despite clear legal and ethical expectations, there is limited knowledge about how assent is experienced by children and their parents within the emotionally charged context of paediatric oncology, in Sweden.

### Aim

The aim of this study was to describe how children with cancer, siblings and parents experience the child’s assent process during research recruitment in paediatric oncology, with particular attention to its motivational and relational dimensions.

## Method

### Design

A descriptive qualitative design was employed [32], with data analysed using reflexive thematic analysis [33]. This present study combines interview material from two independently generated data sets in paediatric oncology. The extended sample was collected specifically to explore experiences of research recruitment and children’s participation in assent. Recruitment of assent-aged children to this sample proved more difficult than anticipated, and only two children were recruited. We therefore considered whether an existing dataset from the Family Talk Intervention (FTI) pilot study could contribute relevant child perspectives. Although generated for a different primary purpose, the FTI child interviews included focused questions concerning who informed the child about the research, opportunities to ask questions, understanding of the research and reasons for participation, and the child’s role and perceived choice in decision-making. These data were therefore considered conceptually relevant to the present research question and included as an additional source of child perspectives. The two datasets were not regarded as equivalent. They differed in research context, participant composition, timing, and depth of data generation. Their combination was based on their shared relevance to the phenomenon under investigation: children’s involvement in research participation and assent within paediatric oncology contexts. The analysis was not designed to establish systematic differences between the datasets or participant groups.

The study forms part of a broader empirical ethics project exploring ethical issues in childhood cancer research recruitment and children’s assent from multiple perspectives.

### Setting

Paediatric oncology care in Sweden is centralized to six regional university hospitals, each hosting a specialised childhood cancer centre. These centres conduct a wide range of clinical, and psychosocial studies involving not only children diagnosed with cancer, but also siblings and parents, reflecting a family-oriented care and research context.

### Recruitment and participants

The two distinct but complementary datasets that this study is based on includes (1) interview data from children diagnosed with cancer and their siblings who participated in a family intervention, and (2) additional interviews with parents and children focusing on participation in clinical studies.

#### Dataset 1: the Family Talk Intervention (FTI)

The first dataset stems from a pilot pre-post intervention study evaluating FTI, a family-based psychosocial intervention, in paediatric oncology [5]. Children diagnosed with cancer (*n* = 9) and their siblings (*n* = 17) were recruited to the FTI by contact nurses at one paediatric oncology centre in Sweden, approximately two to three months after diagnosis or relapse. They were interviewed after the end of the intervention (range: 7–26 weeks; mean: 10 weeks after the intervention ended).

#### Dataset 2: extended sample—interviews on clinical research participation

This sample consisted of parents (*n* = 9) including two parent dyads, and children with cancer (*n* = 2), recruited from a national clinical trial unit and paediatric oncology centres. These participants had experiences with a range of research studies, including psychosocial interventions, precision oncology, and observational research (e.g. biobanking, blood sampling, or genetic studies).

All six paediatric oncology centres in Sweden were invited to assist with recruitment through research nurses and posters/booklets. Five centres participated; one declined due to workload and many ongoing studies. The final sample included participants from four centres, representing southern and central Sweden (the northern region was not represented). The study was also advertised via webpages, social media and newsletters of interest organisations and patient associations.

Across FTI and the extended sample, eligibility criteria included being a child, or parent of a child treated at a Swedish paediatric oncology centre, recruited to research and sufficient proficiency in Swedish. Only children under the age of 15 were included in this study, in line with the legal age for consent in Sweden. For FTI, a subset of interviews with 26 children under the age of 15 was included. In the FTI dataset, families were identified by nurses or research coordinators, and those interested were contacted by an interventionist (social worker, or deacon) who provided age-appropriate verbal and written information. A face-to-face meeting was scheduled with the family in order to inform them about the intervention and the study in more detail. In the extended sample, participants were contacted by a researcher. In total, 37 participants were included: 11 children with cancer, 17 siblings, and nine parents. Participants’ characteristics are presented in Table 2.

**Table 2:** Demographic and clinical data, combined for both datasets (*n* = 37)

	Children with cancer, *n* = 11* N (%)	Siblings, *n* = 17** N (%)	Parents, *n* = 9*** N (%)
Age (years)****			
Mean age	10	10	
Min–max	7–13	5–13	
**Sex**			
Female	6 (55%)	9 (53%)	7 (78%)
Male	5 (45%)	8 (47%)	2 (22%)
**Cancer diagnosis**			
Central nervous system tumour	7 (64%)		
Leukaemia	1 (9%)		
Lymphoma	1 (9%)		
Sarcoma	1 (9%)		
Other	1 (9%)		
**Parental education**			
Upper-secondary	1 (9%)	3 (18%)	2 (22%)
Post-secondary	10 (91%)	14 (82%)	7 (78%)

Upper-secondary: Parent(s) with upper secondary education at most

Post-secondary: At least one parent with post-secondary education at tertiary level (university/higher education) or vocational education

*Nine from FTI and 2 from the extended sample

**All 17 from FTI

***All from the extended sample

****Age data not collected for parents

### Data collection

Across the FTI and the extended sample, data were collected with semi-structured interview guides covering topics such as information and understanding, decision-making and participation (the interview topics for the extended sample are presented in Additional file 1). In the FTI children’s participation in assent was explored through interviews conducted with children after the intervention. These interviews consisted of three focused questions with prompts, embedded within the broader pilot evaluation, and aimed at identifying potential areas for improvement. This included questions about who informed them, their opportunities to ask questions, their understanding of the research and why they had participated, and their role in decision-making (e.g. whether they felt they could choose to participate and whether they had wanted to).

In the extended sample, more in-depth interviews were conducted with both parents and children, focusing on their experiences of recruitment to clinical research. These interview guides were pilot tested with two children and two parents. Only minor revisions were required, and these pilot interviews were included in the analysis. Demographic and clinical background information was collected through questionnaires.

Interviews were conducted at locations chosen by the participants. Child interviews took place at home (*n* = 26) or via video call (*n* = 2). Parent interviews were conducted via video call (*n* = 4) or by phone (*n* = 5). Children were given the option of being interviewed alone or in the presence of a family member (e.g., sibling or parent). Interviews were adapted to the child’s age and communicative abilities. Child interviews lasted on average 15 min (range: 6–29 min), and parent interviews 35 min (range: 26–53 min). All but one parent interview were conducted individually; in one case, both parents participated jointly.

Interviews were carried out by members of the research team (authors KNW and ML, and two doctoral students, see Acknowledgements). None of the interviewers had a prior relationship with the participants or were involved in the children’s clinical care.

Data collection for the FTI pilot took place between September 2018 and August 2019. The extended sample was collected between January 2024 and February 2025. Recruitment of assent-aged children to the extended sample proved substantially more difficult than anticipated, and only two children were included. This recruitment limitation contributed to the decision to include the conceptually relevant child interview material from the earlier FTI study, as described above. The resulting material therefore represents a pragmatic combination of two related but heterogeneous qualitative datasets. All interviews were audio-recorded and transcribed verbatim.

### Data analysis

Interview data were analysed inductively using reflexive thematic analysis, following the six-phase approach outlined by Braun and Clarke [34, 35]. The analysis was led by first author (KNW). The analysis was grounded in critical realism*,* a philosophical framework developed by Roy Bhaskar [36, 37], resting on the assumption that reality objectively exists, but regarding knowledge as interpreted, subjective and context-bound. This analytic approach aimed to identify patterns of meaning related to children’s experiences of being recruited to research, on both manifest and latent levels.

In phase one, *familiarisation*, the first author (KNW) became immersed in the data by reading the transcripts multiple times. This process highlighted shared experiences between children with cancer and siblings regarding participation. The analysis did not seek to compare the views of children with cancer, siblings and parents, but to broadly understand children’s experiences of being invited to participate in research. In phase two, *generating initial codes*, all child interviews were coded using labels that stayed close to participants’ words, focusing on explicit meanings (manifest content). Parent interviews were coded afterwards, and only for content related to their child’s participation in assent and contextually important content, including attitudes towards research. In phase three, *constructing themes*, the first, last and second author (KNW, TG, and SFH) grouped the codes with shared content, across all three participant groups, into preliminary themes and subthemes. This process was iterative, and data driven. In phase four, *reviewing themes*, preliminary themes were refined collaboratively by KNW, TG, and SFH. The team revisited the data to ensure that the themes accurately captured participants’ accounts and that each theme was coherent and distinct. In phase five, *defining and naming themes*, the themes were refined further by KNW who merged, split, or reorganised code groups where appropriately, while revisiting original quotes to ensure anchoring in the data. Themes and subthemes were further discussed, refined and labelled by all three authors. On subtheme-level, themes were kept manifest while the two overarching themes were latent, to meaningfully account for the data, and the contextually relevant moral, motivational and relational aspects narrated. In phase six, *producing the report*, the final themes were written up. Quotes were selected that gave life to the themes, capturing the richness of the children’s voices. Themes were developed through collaborative negotiation in relation to the data, the authors’ theoretical perspectives, clinical experiences, and contextual understanding of the study material. Throughout this phase, thematic interpretations were refined through collaborative discussion among the authors. Through collaborative dialogue, interpretations were critically scrutinised and negotiated in relation to data excerpts, the authors’ theoretical and clinical perspectives and preunderstanding, and the broader ethical narrative developed across the dataset. Reflexivity was woven throughout: in methodological choices, interpretations, and the shared goal of understanding how children and their parents experience the child’s participation when invited into research.

The research team represented several disciplinary and professional perspectives, including psychology, nursing, care ethics, paediatric nursing and physiotherapy, with experiences of research involving children, families, serious illness, and ethical questions concerning research participation and assent. These backgrounds sensitised the analysis to topics such as autonomy, vulnerability, trust, communication, parental responsibility, and children’s participation. At the same time, we recognised that these perspectives could influence which aspects of participants’ accounts we attended to and how we interpreted them.

Data were generated by researchers who were not involved in the children’s clinical care and had no prior relationship with the participants. While this reduced the influence of an established clinical relationship on the interview encounter, the interviewers’ professional and research backgrounds inevitably shaped how questions were asked, followed up, and understood.

Consistent with reflexive thematic analysis, researcher subjectivity was regarded as an integral part of the analytic process rather than as a source of bias that could be eliminated. Developing interpretations were therefore discussed reflexively within the research team, particularly among KNW, TG, ML and SFH. These discussions were used to question initial interpretations, consider alternative readings of the data, and examine how disciplinary, clinical, and ethical assumptions influenced the development of themes. ML’s contextual knowledge of the FTI study and its data generation also informed the interpretation of that material. Collaborative discussion was used to interrogate and enrich the analysis rather than to establish coding consensus or inter-rater agreement.

### Ethical considerations

Families affected by paediatric cancer may experience emotional burden, research fatigue, and dependency on healthcare professionals. To minimise undue pressure, families in acute crisis were not approached. Those invited to participate were given sufficient time to consider participation independently and without time pressure. A referral pathway to appropriate psychosocial support services was established should participation evoke distress. Voluntariness was emphasised throughout the study. Children were provided with age-appropriate information, and their explicit assent or dissent was actively sought. The interviewer remained attentive to verbal and non-verbal signs of discomfort or reluctance. When children chose to be interviewed in the presence of a parent, it was clarified that the interview focused on the child’s own perspective.

Data were collected and managed to protect participants’ privacy, through pseudo-anonymisation, password-protected interviews, and results presented at group level. Data were handled in accordance with General Data Protection Regulation (GDPR) and Uppsala University’s internal regulations. All data were securely stored in encrypted form, in compliance with applicable information classification standards. Given the small and closely connected context of Swedish paediatric oncology care, and to protect participants’ confidentiality, the specific type of research each participant was involved in has been excluded in the results presentation. All quotes are attributed using pseudonyms.

## Results

### Theme 1: a moral and emotional commitment to research

When children, siblings and parents reflected on recruitment to research, their responses were often positive. Participation was described not merely as a practical decision but as morally and emotionally meaningful. Research was framed as an act of solidarity, contributing to the “greater good”, and as a source of comfort and purpose in an otherwise challenging situation.

#### Giving back and paying forward

Children, siblings and parents expressed a clear awareness of the importance of research for improving childhood cancer care. Many described a strong sense of moral responsibility to contribute, alongside a sense of meaning in being able to help others facing similar circumstances.

I wanted to help others... so that things would be better for them. *(Maria, child with cancer, 12 years)*

Parents evaluated research invitations in light of deep gratitude for the care their child had received. Participation was described as a way of reciprocating previous families’ contribution and “giving back” to the healthcare system. In some cases, this gratitude made declining participation feel almost unrealistic.

I don’t think many people would say no when they have saved their child’s life. *(Mats, parent of Adrian, child with cancer, 10 years)*

#### Unquestioned participation for the greater good

For many families, agreeing to participate in research was described as self-evident. Several expressed a general, predetermined willingness to accept all research invitations, rather than evaluating each study individually.

We’re involved in all research, as a help… all the research we are allowed to take part in. *(Arvid, child with cancer, 8 years)*

The willingness to participate was often grounded in a profound trust in healthcare professionals and in the perceived integrity and inherent value of research. Participation was often framed as inherently beneficial. This was frequently accompanied by an unreflective approach to what participation actually entailed for the child. The need to understand details or question potential risks was seen as secondary—or even unnecessary.

I didn’t care at all what it was, I knew it would be for something good anyway. *(Adrian, child with cancer, 10 years)*

Research whatever you want, as long as you… make the world a better place and fewer children get sick… If we get listed in systems and it's GDPR [General Data Protection Regulation] or... I mean, completely uninteresting!... I can’t explain at all what it was that they were supposed to do research on. *(Kristina, parent of Silje, child with cancer, 14 years)*

#### Feeling cared for and supported

Research participation was also described as providing opportunities for care and emotional support. For some children, being invited to participate created a sense of being acknowledged, chosen, or seen, which added a positive emotional dimension to the otherwise difficult experience. Participation was not only about contributing to knowledge but also about being given space to express thought and feelings.

They asked me... if I wanted to, and I did, because I think it feels good to talk to someone about [name of sibling with cancer]'s cancer. *(Thilde, sibling, 8 years)*

Children, particularly siblings, valued the opportunity to talk with someone outside the immediate family about their experiences. Research encounters were sometimes experienced as supportive conversations rather than solely data collection.

Parents of children with cancer similarly described research as offering an additional layer of attention and follow-up. In some cases, participation was perceived as providing a level of care, continuity, or emotional support that they felt was not always fully met within standard childhood cancer care.

In a study... you get more check-ups. I mean, to check for side effects and stuff, you get scanned more... they don’t just let him go without control, he’s been very closely monitored... they’re very responsive... and we always have someone to call. I mean, around day and night. *(Camilla, parent of Matteo, child with cancer, 13 years)*

#### Not at any cost: ambivalence and boundaries

Despite the overall willingness to contribute to research, children, siblings and parents expressed reservations and boundaries. Participation was not unconditional. For some children, the research topic did not feel personally relevant, which reduced their motivation to participate. Others described a wish to protect their personal space and avoid sharing sensitive experiences with researchers.

At first, I felt negative about it because I didn’t want to. I don’t like talking to people about what happened. *(Emil, sibling, 11 years)*

Parents also voiced concerns about the potential impact of participation on the child, emphasizing their moral responsibility to protect their child from additional burdens.

I mean, number one was that this would not affect [him] at all. It shouldn’t add a single burden on him whatsoever... Not a single test or anything extra for him, he shouldn’t have to... he has enough as it is. That was kind of the most important... as long as he doesn’t have to do anything, that it’s hidden in his treatment. *(Ann-Sofie, parent of Oscar, child with cancer, 8 years)*

Despite these concerns, many families ultimately chose to participate, finding reassurance in the belief that research served a higher purpose and in their trust in the integrity of research and the good intentions of researchers.

Overall, this theme shows that research was widely viewed as relevant and meaningful among families in paediatric oncology. It highlights how moral, motivational, and relational motives play a central role in the decision to participate, serving the backdrop in which children’s assent takes place. Not least how solidarity, gratitude, trust, safety and support may be more decisive for families than scrutinising individual research studies in detail.

### Theme 2: relational assent—trust, loyalty and parental mediation

Children’s motivation to participate in research and their decision-making process were closely shaped by their relationships with parents. Trust, loyalty, and mutual protection influenced how assent unfolded, and children’s involvement varied depending on parental practices and emotional strain. Rather than an individual or isolated choice, agreeing to research participation was often described as a relational process, shaped by children’s sensitivity to parental expectations and emotional needs.

#### Mutual protection

In parents’ accounts of the informed consent process, a clear sense of vulnerability emerged. Being approached about research while the family was navigating a life situation marked by trauma and stress was described as emotionally demanding. In this context, parents univocally emphasised a moral responsibility to protect their child—from harm, from overwhelming or distressing information, and from additional strain, by assuming the role of decision-maker and primary source of hope and emotional support.

There is no psychologist linked to [Tristan] in a research study, so I think it’s up to us parents to also be a psychologist for our child, who is in a difficult situation and has lived in a difficult situation for a long time. So, we were more like, now [Tristan], you’re going to get tablet treatment instead of this strong one, you know, that makes you feel sick all the time. We have to be positive and hopeful, that’s our responsibility. (*Karin, parent of Tristan, child with cancer, 9 years*)

Parents described that their child sometimes withheld concerns, for example about research, not to burden or worry them—pointing to a pattern of mutual emotional protection between child and parent. When asked to describe the assent process, children often times reported receiving information about the research primarily through their parents, especially when the study team was not based at the child’s hospital. This reinforced the parents’ role as mediators and gatekeepers of both information and participation.

#### Trust and alignment with parents

Children expressed great trust in their parents’ judgement and a conviction that parents knew what was best for them—describing it as comforting and reassuring to follow their parents’ suggestions. This trust among children sometimes meant that they did not actively reflect on research participation as a personal choice, or felt a need to gain an understanding of what it entailed.

They asked me if I thought it was a good idea and I tagged along, because it’s like… if mum and dad think it’s good, then I think it’s going to be all right too. (*Arvid, child with cancer, 8 years*)

Parents mirrored this experience, while also noting that their child’s reliance on their judgement could sometimes undermine the child’s own involvement, leaving them uninformed. Over time, this raised ethical concerns for some parents about how the decision had been made and whether it was legitimate.

He has been really confident and safe with us parents deciding, but it’s only now that he’s started to ask questions, like when will my treatment end, and then I’ve thought about it – well, he’s not involved in the decisions. No, he’s not. *(Karin, parent of Tristan, child with cancer, 9 years)*

#### Assent motivated by loyalty

Some children described that they said yes to research to avoid disappointing their parents or because they perceived the research as important to them. In those cases, assent constituted an act shaped primarily by loyalty, where the child prioritised their parents’ feelings and wishes, regardless of their own internal motivation.

Mum couldn’t have forced us if I had refused… but I mean, she would have been upset. If I had refused… or, you know, I knew that mum thought it was important, so… yes. I did it… I feel like I kind of did it for her sake [laughs]. *(Ella, sibling, 12 years)*

#### Pre-determined decisions

Children sometimes described situations in which the decision to participate had already been made by their parents, without their involvement. When informed by their parent, the conversation was framed as if research participation was already decided, rather than presented as something open to the child’s consideration.

No, they had already decided. *(Alva, sibling, 13 years)*

Some children could not recall being informed by parents beforehand about what the research entailed, and participation was unexpected.

They didn’t even tell me. All I knew was that two people were coming to talk to me. [Interviewer: Was that so? Who told you that two people were coming?] Mum... That was all I knew. *(Samuel, sibling, 9 years)*

Parents sometimes also described a lack of direct engagement with their child from researchers. One father, for instance noted that no tailored information was given to his 13-year-old daughter, even though she was present during the conversation. Importantly, none of the children expressed resistance or questioned their parents’ decision. Rather, they described the process in neutral terms, acknowledging how decisions had been made on their behalf.

#### Parental influence

In some cases, the children expressed that they initially did not want to participate, or were hesitant, but agreed after repeated conversations and encouragement from their parents. This suggests that parents’ desire to be part of research could create subtle forms of influence on the child.

At first, I said I really didn’t want to and then mum and dad said okay, but the next day they asked if I was really sure about it and then I said I could try. *(Emil, sibling, 11 years)*

Overall, children expressed satisfaction with participating and that they had wanted to take part. Some children reflected that they might have declined research participation if they had felt entirely free to choose, indicating a perceived limitation of agency and self-determination. However, their accounts were often nuanced—although they had felt expectations from their parents to take part, parental influence was not necessarily perceived as something inappropriate to the child.

#### Mental load as a barrier

Children’s limited role in research assent was at times self-chosen, due to lack of interest or energy—or, in the case of children with cancer, because they were emotionally overwhelmed and mentally unavailable particularly in the immediate aftermath of diagnosis.

At first [Maria] didn’t talk much, which is understandable... it was enough, everything she was going through... why me, and then having to deal with all this paperwork. You wish they could’ve waited a bit, not right at the beginning when so much is happening, and you [referring to the child] don’t feel like doing anything or caring about anything, you’re just angry. *(Sonya, parent of Maria, child with cancer, 13 years)*

A parent described how the son’s psychological distress made even minimal interaction impossible.

They couldn’t say a word to him. When they came in, he just screamed… he was in such a bad mental state, so incredibly bad, that there was no alternative, and we’re still convinced he wasn’t mentally present. *(Ann-Sofie, parent of Oscar, child with cancer, 8 years)*

Parents noted that once their child had “settled” emotionally, they became more receptive to communication. However, no renewed effort was made by researchers to involve the child or explain their research participation. This surprised parents, who viewed it as a missed moral responsibility.

No one has really talked to him about the research or explained it. I think it’s wise to talk to children, maybe not at the beginning… but a bit later… Like: ‘I just want to explain a bit more, your mom and dad agreed to research that’s really important, but we also want to explain to you what it means to be part of research and talk about the study you’re involved in,’ and so on. Because it’s also about the future. I mean, showing a bit about what a hospital does and why treatments get better, and that sometimes it’s important to be a participant. *(Anna, parent of Adrian, child with cancer, 10 years)*

#### Unclear and disconnected participation

In some cases, children’s limited role in assent was linked to confusion or lack of awareness about their involvement in research. They expressed uncertainty about the research they had participated in, such as the purpose of the study and their role in it.

I honestly have no idea [what research I’ve been part of]. *(Adrian, child with cancer, 10 years)*

With time passing without renewed engagement, recall of the research experience became vaguer and could make families feel adrift and disconnected to their research experience.

The thing is, because we don’t get any follow-up or feedback, you don’t get any kind of connection to... what the heck they were supposed to be researching? I mean, I don’t go around thinking about this, ‘aha, I wonder if they’ve gotten anywhere’, because I don’t even remember what it was about. *(Kristina, parent of Silje, child with cancer 14 years)*

Overall, this theme illustrates how children’s assent was shaped by relational and emotional dynamics within the family. It highlights the role of trust, loyalty, dependency, and mutual care for children’s involvement.

## Discussion

This study describes children, siblings and parents’ experiences of the child’s assent process in paediatric oncology research, with particular attention to its motivational and relational dimensions. Overall, participation in research was perceived as meaningful and morally significant, underpinned by strong trust in the value and integrity of research. At the same time, children’s understanding and active involvement in decision-making were sometimes limited, particularly in emotionally demanding situations. Children’s engagement was strongly shaped by relational dynamics with their parents.

Our findings align with previous research showing that children with cancer, their siblings, and parents often experience psychosocial benefits from research participation, such as feeling cared for and supported [28, 30, 38]. The results also reflect discourses within paediatric oncology that emphasise the moral value of contributing to research as a way of giving back [39]. At the same time, the trust and partnership with families that are fundamental to sustainable research practice [40] may also undermine critical reflection on the potential risks and burdens of research [7, 39], as highlighted in this study. Understanding the context in which children’s participation in assent takes place, also in terms of motivational and relational motives, is essential. Not least because meaningful assent requires not only considering the child’s age and maturity but also their individual circumstances and family situation [8].

Our findings further confirm that, despite ethical guidelines requiring meaningful child assent [8], children’s participation in assent may be limited by several factors [21, 22], including emotional distress and cognitive conditions. In this study, many children had CNS tumours, a diagnosis often associated with cognitive impairment [41, 42]. The child’s clinical status is important to consider, as it may influence the child’s capacity to engage in research discussions as well as parents’ and researchers’ attitudes towards involving the child in decision-making.

Previous research has shown that professionals’ and parents’ motivation to protect the child may gatekeep children’s access to information and opportunities to participate in decisions [23, 26, 43]. In this study, protection emerged as a relational dynamic in which parents sought to shield the child from potentially burdensome information and decisions, while children expressed loyalty and trust towards their parents. These findings highlight the need for inclusive participation practices that respect children’s capacities and preferences for involvement, while safeguarding their wellbeing and vulnerability [15].

Our results are consistent with relational perspectives on autonomy, which emphasise that agency is socially embedded and shaped through relationships that may both enable and constrain it [44, 45]. This is particularly relevant in paediatric contexts, where children’s decision-making takes place in close interaction with parents and professionals [46].

The findings in this study illustrate how children’s participation in the assent process is often mediated through their parents, which may be preferred and reassuring for the child, but how trust in parents may also limit children’s involvement and understanding of research. This may be a particular ethical challenge when participation is based on a therapeutic misconception, where families believe that the study is intended to directly improve the child’s treatment or care [7, 47, 48].

Meaningful participation requires responsibility, knowledge, and skills among researchers [15]. Our findings underscore the importance of relational awareness in promoting ethically robust research recruitment [7, 49, 50]. Our results further suggest that parental influence may create subtle pressure or loyalty conflicts for the child, particularly when research participation involves the entire family, as in FTI. Since research is generally not designed to directly benefit the individual child, children should be supported in declining participation, in situations where it feels burdensome or intrusive [51–53]. Researchers therefore have an ethical responsibility to safeguard children’s integrity and right to refuse participation in practice, ideally in collaboration with parents [54]. This requires relational awareness of how, in each family, the parents’ attitudes and behaviours shape their child’s participation in research and assent, as well as tailored support, including procedural, emotional, and psychosocial support.

Assent should be understood as a process rather than a single event, implying ethical responsibility over time [15]. This requires attention to, and distinguishing between, (1) initial assent and (2) children’s ongoing opportunities to understand, reconsider, or withdraw their participation. Here, our findings indicate a gap in practice: children with cancer who were initially too ill or emotionally overwhelmed to meaningfully engage in the assent process were rarely re-approached later in the treatment trajectory, despite improved conditions and capacity. It was this absence of ongoing involvement, in particular, that parents highlighted as a missed moral opportunity. The distinction between initial assent and ongoing involvement highlights the need for situated and dynamic models of assent that accommodate children’s changing abilities and needs over time [55, 56], as well as ongoing ethical reflection on children’s participation throughout the entire research process, not only during recruitment [57, 58]. A situated and dynamic approach appears particularly important in paediatric research, where the conditions for children’s involvement and families’ circumstances may vary considerably across different stages of the illness trajectory. Ethical conduct, in this view, is not a checklist but an ongoing practice that also acknowledges that children’s agency and need for protection coexist and must be wisely integrated in each situation [16]. Such an approach may help avoid two extremes: an overemphasis on protection that marginalises children’s perspectives and undermines meaningful participation, or an exclusive focus on autonomy that may expose children to other forms of vulnerability and risk [59, 60].

### Clinical implications

Our findings highlight several considerations for research practice. Children’s and parents’ willingness to participate should not be assumed to indicate that meaningful assent has occurred, as positive attitudes towards research *may* coexist with limited information, understanding, or involvement in decision-making. The findings also highlight the central role of parents, whose involvement may both support and constrain children’s participation, and support understanding assent as an ongoing rather than a one-term process.

To support meaningful child assent, previous research has proposed repeated dialogue, relational sensitivity, and flexible communication strategies that accommodate the child’s changing cognitive, emotional, and psychological states, throughout treatment trajectory [61–64]. A lack of practical guidance and harmonisation across settings and jurisdictions remains a recognised challenge [65, 66]. Conceptual frameworks such as the PAeDS-MoRe framework [67] together with flexible and interactive tools [64, 68], participatory visual methods [69], narrative recall approaches [70] and dialogue-based techniques [71] may support the development of more responsive assent processes. Further methodological development is particularly needed for younger children. In addition, the distinct position of siblings [72] as potential research participants warrants greater recognition and exploration.

Given parents’ central role in mediating children’s involvement in assent, they should be understood not only as legal decision-makers, but as ethical partners in the research process. Their knowledge of the child and their emotional engagement can strengthen ethical integrity in assent procedures, provided that their role supports rather than overrides the child’s voice [47, 54, 73]. Such shared engagement may provide a solid foundation for meaningful child participation in assent, although further research is needed to determine how best to operationalise this in paediatric research.

### Strengths and limitations

A strength of this study is the focus on children in the assent age-appropriate range, a population often under-represented in empirical ethics research. The primary focus was on the broader ethical dimensions of recruitment and assent rather than context-specific considerations, although they remain important. Further, from a research ethics perspective, the requirement for assent prior to research equally applies irrespective of study design and whether the participant is an ill child or a sibling. By including perspectives from children with cancer, siblings, and parents, the study illuminated relational and contextual aspects of assent and provided a nuanced understanding of the process.

Several limitations should also be considered. First, the transferability of the findings may be limited as all participants were Swedish-speaking and the majority of parents had higher education, potentially limiting socioeconomic and cultural diversity in the sample. Second, the study does not include perspectives from families who declined research participation, who may have held more critical views of research involvement. The prominent themes of trust, moral responsibility, and willingness to volunteer identified in this study should therefore be interpreted with caution, as previous research suggests a more nuanced picture, reporting reluctance and distrust among families, related to concerns about confidentiality, authorities, and fear of experimentation [7]. Third, the two datasets differed in study type, recruitment context and participant composition, and this may have obscured context-specific patterns. For example, participation in a psychosocial family intervention may involve different motivations, expectations, and relational dynamics than participation in clinical or biomedical research. Similarly, children’s involvement may be influenced by who introduces and discusses the research with the family, as well as by participant characteristics such as age, diagnosis, health status, and whether the participant is a child with cancer or a sibling. The present analysis was not designed to examine such differences systematically, and the findings should therefore not be interpreted as demonstrating equivalence across research contexts or participant groups.

Additional limitations arise from the retrospective nature of the interviews. In some cases, several weeks had passed between the child’s research participation and the interview (range: 7–26 weeks, mean 10 weeks), which did affect recall accuracy and introduced recall bias, particularly among younger children or those who had experienced high emotional distress. Children could not always recall the extent of their involvement, the information they received, or how decisions were made. Parents’ accounts provided an additional perspective on the assent process, helping to contextualise and corroborate children’s accounts. Furthermore, data collection regarding assent within the FTI study was limited to three focused questions embedded within a broader pilot evaluation, limiting opportunities for a more in-depth exploration of children’s perspectives, and discerning important nuances, for example regarding *supportive* versus *constraining* parental mediation. Due to recruitment challenges, only two children were interviewed in the extended sample. Restrictive eligibility criteria requiring assent-aged children with recallable experiences of research participation (excluding children with early childhood tumours and older teenagers), and children being able to recall and meaningfully reflect on experiences of research participation limited the pool of eligible patients. Parents were often more willing to participate than children, potentially due to illness-related vulnerability [63]. Further, children may have limited recollection of research and assent, and not all children are involved in research discussions or assent [21, 22], and research may be an abstract topic to talk about [54]. Despite these limitations, the study contributes valuable insights into how children and families experience research assent processes and highlights the significance of inclusive, child-centred recruitment practices and relational awareness in paediatric research.

## Conclusions

Families’ high willingness to contribute to research is grounded in a strong moral commitment to reciprocate previous families’ contributions and to help improve care for future children. Maintaining trust and research integrity requires consistent and meaningful realisation of children’s own assent to research, particularly given the dominant role parents may play in shaping children’s participation and autonomy in research decisions. This presupposes child-centred, situated and dynamic assent procedures, and recognition of parents as key partners in upholding children’s rights and ethical standards in paediatric oncology research.

## Supplementary Information

Additional file 1. Interview topics in the extended sample, related to children’s participation in research assent, with examples questions.

## Data Availability

Due to privacy and ethical restrictions, data are not publicly available. Anonymous data that support the findings are available upon reasonable request to the corresponding author.
