# Supplementary material for: Children’s participation in assent to paediatric oncology research: experiences of children, siblings and parents
Source: Trials. 2026 Sep 21;27:579. doi: 10.1186/s13063-026-10011-2 (PMC13591860; doi:10.1186/s13063-026-10011-2)
Supplement: Supplementary file 1 [file 13063_2026_10011_MOESM1_ESM.docx]

**Additional file 1**. Interview topics in the extended sample, related to children’s participation in research assent with examples questions.

| Children with cancer | Parents of children with cancer |
| --- | --- |
| **Information and understanding** | **Information and understanding** |
| You've been involved in research, right? I'm curious to know what you thought and felt when they asked you to participate.  How did it happen, did the researcher ask you or your parents or both about the study?  *With additional prompts like;*  Did the researcher tell you about what the research was about? Did you understand what the researcher said? Was there anything you found difficult? | Tell me about when you were asked if your child could participate in research, what you thought and felt about being asked.  *With additional prompts like;*  Did the child get any information? Concerning information, what was especially important for you as a parent? Was something difficult? What do you think is important for researchers to consider when informing families where the child has cancer about studies? |
| **Participation in decision-making** | **Participation in decision-making** |
| How was it decided that you would participate?  *With additional prompts like;*  Who decided that you would participate? Were you able to express your opinions, thoughts and wishes? Did you want to participate in the research? How come? Could you have said no if you didn't want to? Was it important to be able to participate in the decision-making process, or was it better that they decided?  What did you think about the fact that you were going to participate? Was it good? Bad? In what way? Was there anything you particularly liked or disliked about participating? Could you talk to the researcher or your parents about it? | Could you tell me a little more about how it was decided that [child's name] should participate?  *With additional prompts like*;  What made you decide that it was okay for the child to enroll?  Was the child involved, or was it more you as parents who took responsibility for the decision? What do you think about it being that way?  When families are asked if their child can participate in research, what role do you think is appropriate and good for children to have? |
| **General reflective questions** | **General reflective questions** |
| If you were to tell a friend (who also has cancer) about what it’s like to be asked to participate in research, what would you say?  *With additional prompts like*; Was there anything you liked or thought was good? Was there anything you would have liked to be different so that it would have been better, more fun or easier for you?  What advice would you give to researchers who ask children/teenagers with cancer to participate in research? | How would you describe to another parent of a child with cancer what it is like to be asked about research?  *With additional prompts like*;  Would it have been good to have more support from the researcher or for the researcher to have done something differently?  Based on your experience as a parent, do you have any advice for researchers when it comes to asking children with cancer and their parents about research? |
